# A new species and new records of the leafhopper genus *Taperus* Li & Wang, 1994 (Hemiptera,Cicadellidae, Evacanthinae) from China

**DOI:** 10.3897/zookeys.120.1373

**Published:** 2011-07-25

**Authors:** Yu-jian Li, Zi-zhong Li

**Affiliations:** 1 Institute of Entomology, Guizhou University, Guiyang, Guizhou Province 550025, China; 2 The Provincial Key Laboratory for Agricultural Pest Management of Mountainous Region, Guizhou University, Guiyang, Guizhou Province 550025, China

**Keywords:** Auchenorrhyncha, morphology, identification, China

## Abstract

The paper deals with the species of the Oriental leafhopper genus *Taperus* Li & Wang. A new species, *Taperus daozhenensis* **sp. n.,** from Guizhou Province, China is described and new records for other Chinese species are given together with a key for their separation. The type specimens of the new species are deposited in the Institute of Entomology, Guizhou University (GUGC).

## Introduction

The leafhopper genus *Taperus* was established by Li and Wang in 1994 with *Taperus fasciatus* Li & Wang as its type species, at same time Li and Wang described two more species, *Taperus albivittatus* and *Taperus apicalis*. Subsequently, three new species were described in the genus by (Cai and Shen (1997, 1999) and Li and Wang (2001). Zhang et al. (2010) reviewed the genus and described three new species from China in addition to transferring one species from *Onukia* to *Taperus* and three species from *Taperus* to the genus *Convexana* Li.

A new species from Guizhou Province, China is here described and illustrated. A key to all known species of *Taperus* is given and new records for other Chinese species of the genus are provided. Currently eight species are included in the genus.

The specimens used in this study are deposited in the Insititute of Entomology, Guizhou University, Guiyang, China (GUGC).

## Taxonomy

### Taperus

Li & Wang

http://species-id.net/wiki/Taperus

Taperus Li and Wang 1994: 374

#### Type species.

 *Taperus fasciatus* Li & Wang.

#### Diagnosis.

 *Taperus* is similar to *Onukia* Matsumura, it differs from this genus in having the median longitudinal carina of vertex very weak, nearly indistinct; area between median carina and submarginal carina of vertex nearly flat rather than concave and male pygofer with rows or groups of stout spine-like macrosetae marginally.

For detailed generic description see Zhang et al. (2010).

#### Key to species of the genus *Taperus* Li & Wang based on male adults

**Table:** 

1	Aedeagal shaft with a triangular posterior process in lateral view; foot-like apical process of style with apex very long and slender	2
–	Aedeagal shaft not or slightly developed posteriorly near base in lateral view; foot-like apical process of style with apex short and robust	4
2	Male pygofer longer than wide, with apical half distinctly narrowed in lateral view	3
–	Male pygofer nearly discoidal in lateral view	*Taperus apicalis* Li & Wang
3	Male pygofer with a row of long spine-like macrosetae on dorsal margin	*Taperus bannaensis* Zhang, Zhang & Wei
–	Male pygofer with short thick macrosetae at apex	*Taperus fasciatus* Li & Wang
4	Male pygofer with apex truncate in lateral view	5
–	Male pygofer distinctly narrowed apically with apex rounded in lateral view	6
5	Male pygofer somewhat rectangular in lateral view; style elongate	*Taperus flavifrons* (Matsumura)
–	Male pygofer nearly quadrangular in lateral view; style short	*Taperus quadragulatus* Zhang, Zhang & Wei
6	Apex of aedeagus with processes	7
–	Apex of aedeagus without processes	*Taperus luchunensis* Zhang, Zhang & Wei
7	Apex of aedeagus with three spine-like processes on dorsal margin and four pairs of spine-like process on ventral margin	*Taperus albivittatus* Li & Wang
–	Apex of aedeagus with two retrorse spine-like processes on dorsal margin and one big spine-like process on dorsal margin subapically (Figs 4, 5)	*Taperus daozhenensis* sp. n.

### Taperus albivittatus

Li & Wang

http://species-id.net/wiki/Taperus_albivittatus

Taperus albivittatus Li and Wang, 1994: 375; 1996: 115; Zhang et al. 2010: 39

#### Distribution.

China (Sichuan).

#### Hosts.

Unknown.

#### Material examined.

1♂(Holotype): CHINA, Sichuan: Emeishan, Wanniansi, 3 August 1991, coll. Li Zizhong.

#### Notice.

After reexamining the holotype of this species, we found the count of spine-like processes on the apex of the aedeagus was wrong in the original description, we corrected it in above keys.

### Taperus apicalis

Li & Wang

http://species-id.net/wiki/Taperus_apicalis

Taperus apicalis Li and Wang 1994: 377; 1996: 116; Zhang et al. 2010: 39

#### Distribution.

China (Guizhou).

#### Hosts.

Unknown.

#### Material examined.

1♂(Holotype): CHINA, Guizhou: Shuicheng, 30 September 1987, coll. Li Zizhong.

### Taperus fasciatus

Li & Wang

http://species-id.net/wiki/Taperus_fasciatus

Taperus fasciatus Li and Wang 1994: 378; 1996: 117; Zhang et al. 2010: 39

#### Distribution.

China (Guizhou, Hainan, Guangxi, Sichuan, Fujian, Shaanxi, Hunan, Zhejiang, Jiangxi); Vietnam.

#### Hosts.

Unknown.

#### Material examined.

1♂(Holotype): CHINA, Guizhou: Daozhen, 18 September 1988, coll. Li Zizhong; 20♂♂, 17♀♀: CHINA, Guizhou: Leigongshan, Xiaodanjiang, 13~14 September 2005, coll. Li Zizhong & Zhang Bin; 6♂♂7♀♀: CHINA, Guizhou: Kuankuoshui, Chachang, 10~17 August 2010, coll. Dai Renhuai, Li Hu & Fan Zhihua; 16♂♂7♀♀: CHINA, Guizhou: Kuankuoshui, Chachang, 14~17 August 2010, coll. Yu Xiaofei; 3♂♂: CHINA, Guizhou: Kuankuoshui, Chachang, 12 August 2010, coll. Li Yujian; 2♂♂: CHINA, Hainan: Jianfengling, 10~12 July 2007, coll. Li Yujian; 2♂♂: CHINA, Guangxi: Huaping, 11 June 1994, coll. Du Yuzhou; 1♂: CHINA, Zhejiang: Longquan, 17 June 1980, coll. Tong Xuesong; 3♂♂: CHINA, Sichuan: Baishuihe, 27 August 2007, coll. Xing Jichun.

### Taperus flavifrons

(Matsumura)

http://species-id.net/wiki/Taperus_flavifrons

Onukia flavifrons Matsumura, 1912: 45
Taperus flavifrons (Matsumura); Zhang et al. 2010: 39

#### Distribution.

China (Hainan, Taiwan); Japan.

#### Hosts.

Unknown.

#### Material examined.

2♂♂: CHINA, Hainan: Wuzhishan, 13~15 July 2007, coll. Zhang Bin; 1♂: CHINA, Hainan: Wuzhishan, 13~15 July 2007, coll. Li Yujian; 1♂: CHINA, Hainan: Diaoluoshan, 16~18 July 2007, coll. Zhang Bin; 1♀: CHINA, Hainan: Wuzhishan, 13~15 July 2007, coll. Zhang Hui; 1♀: CHINA, Hainan: Wuzhishan, 13~15 July 2007, coll. Song Yuehua; 1♀: CHINA, Hainan: Diaoluoshan, 16~18 July 2007, coll. Zhang Hui; 1♀: CHINA, Hainan: Diaoluoshan, 16~18 July 2007, coll. Zhang Bin.

### Taperus bannaensis

Zhang, Zhang & Wei

http://species-id.net/wiki/Taperus_bannaensis

Taperus bannaensis Zhang et al. 2010: 44

#### Distribution.

China (Yunnan, Hainan).

#### Hosts.

Unknown.

#### Material examined.

1♂: CHINA, Hainan: Limuling, 23 May 1997, coll. Yang Maofa.

### Taperus quadragulatus

Zhang, Zhang & Wei

http://species-id.net/wiki/Taperus_quadragulatus

Taperus quadragulatus Zhang et al. 2010: 44

#### Distribution.

China (Hunan, Guizhou).

#### Hosts.

Unknown.

### Taperus luchunensis

Zhang, Zhang & Wei

http://species-id.net/wiki/Taperus_luchunensis

Taperus luchunensis Zhang et al. 2010: 45

#### Distribution.

China (Guizhou, Hubei, Yunnan).

#### Hosts.

Unknown.

#### Material examined.

4♂♂, 5♀♀: CHINA, Guizhou: Leigongshan, Xiaodanjiang, 13~14 September 2005, coll. Li Zizhong & Zhang Bin; 1♂: CHINA, Hubei: Shennongjia, 13 August 2004, coll. Peng Jingyang.

### Taperus daozhenensis sp. n.

urn:lsid:zoobank.org:act:35578DE4-6CF7-4389-B007-F306E375AE90

http://species-id.net/wiki/Taperus_daozhenensis

Figs 1 –7 

#### Measurements.

 ♂: body length: 6.0 mm; head width (incl. eyes): 1.1mm; head length: 0.8mm.

**Figures 1–2.**
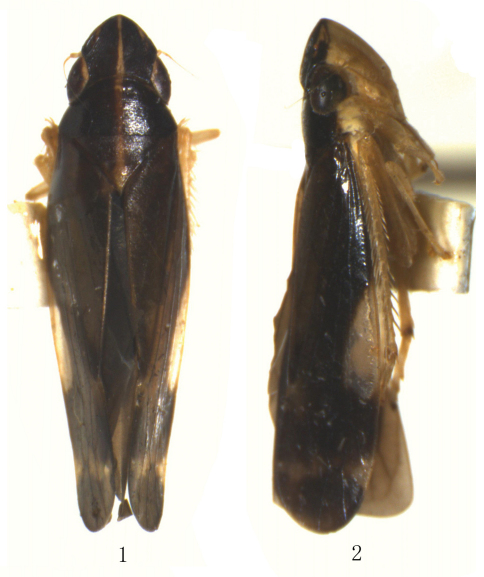
*Taperus daozhenensis* sp. n. **1–2** Male, dorsal view and lateral view.

Vertex, pronotum and scutellum dark brown; pale median longitudinal band yellow, extending from apex of vertex to posterior margin of pronotum (Figs 1, 2). Face nearly pale yellow (Figs 1, 2). Forewing (Figs 1, 2) dark brown, with nearly white and transparent plaque in middle of costal area.

**Figures 3–7.**
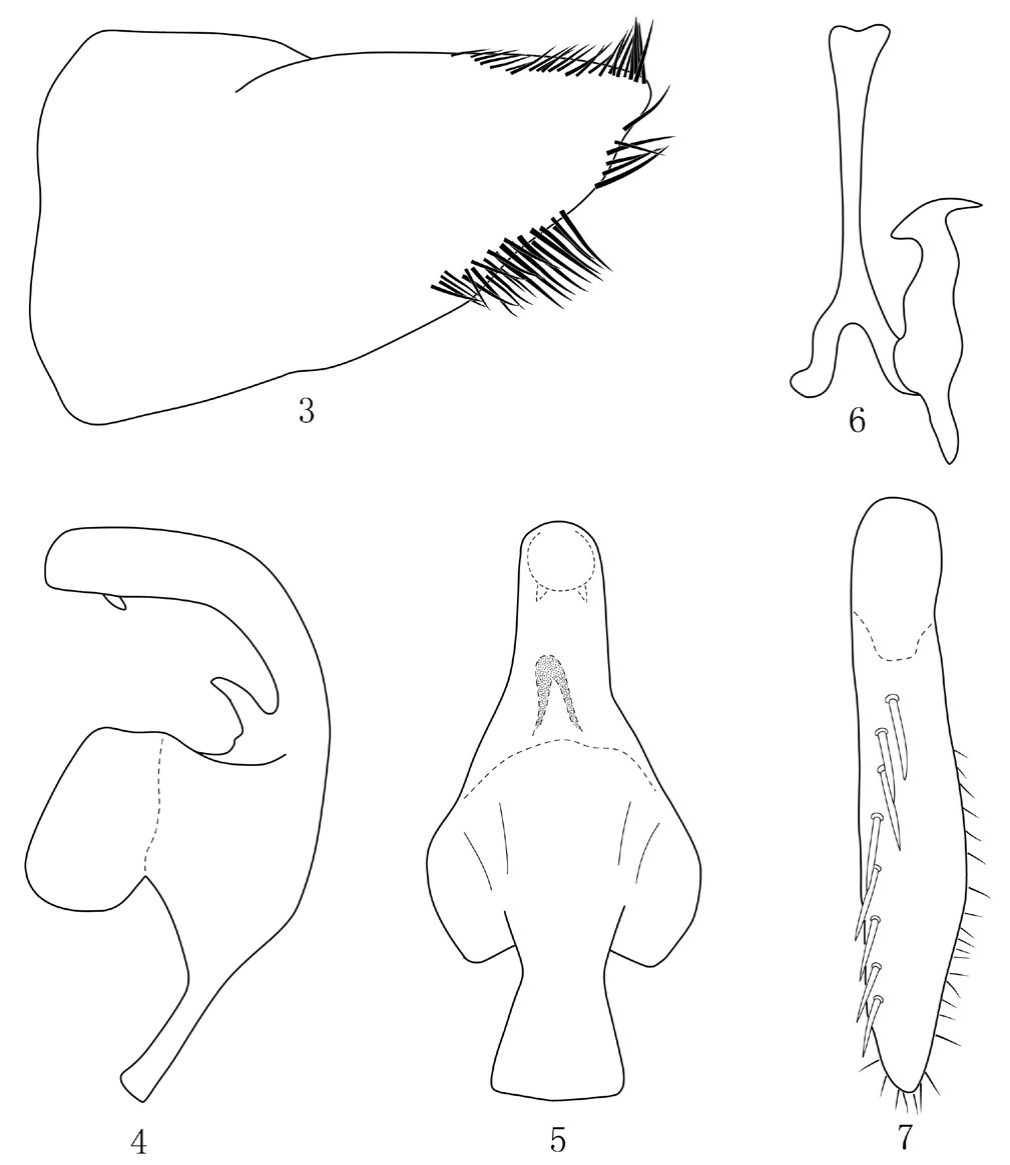
*Taperus Daozhenensis* sp. n. **3** Male pygofer, lateral view **4** Aedeagus, lateral view **5** Aedeagus, ventral view **6** style and connective **7** Subgenital plate.

Male pygofer (Fig. 3) nearly triangular in lateral view, row of long macrosetae at end of dorsal and ventral margin. Apex of foot-like apical process of style very long and slender, about one third length of style (Fig. 6). Aedeagal shaft, recurved dorsally, expanded distally in lateral view, broadly rounded apically with pair of retrorse spine-like processes on dorsal margin subapically and one big spine-like process on dorsal margin near base (Figs 4, 5).

Other characteristics are as shown in Figs 1–7.

#### Type Material.

Holotype: ♂, China: Guizhou: Daozhen, 17~22 August 2004, coll. Yang Maofa; Paratypes: 2♂♂, China: Guizhou: Daozhen, 17~22 August 2004, coll. Yang Maofa.

#### Etymology.

The species is named after the locality of the type specimens, Daozhen county.

#### Remarks.

This species resembles *Taperus albivittatus* Li & Wang, from which it is distinguished by: aedeagus with pair of subapical retrorse spine-like processes dorsally and one big spine-like process on dorsal margin near base. Apex of aedeagusof *Taperus albivittatus* has three spine-like processes on dorsal margin and four pairs of spine-like process on ventral margin.

## Supplementary Material

XML Treatment for Taperus

XML Treatment for Taperus albivittatus

XML Treatment for Taperus apicalis

XML Treatment for Taperus fasciatus

XML Treatment for Taperus flavifrons

XML Treatment for Taperus bannaensis

XML Treatment for Taperus quadragulatus

XML Treatment for Taperus luchunensis

XML Treatment for Taperus daozhenensis
